# Mini-percutaneous nephrolithotomy via ultrasound guidance in transplanted kidney: a report of two cases

**DOI:** 10.11604/pamj.2022.41.333.33169

**Published:** 2022-04-26

**Authors:** Ali Eslahi, Mehdi Salehipour, Mohammad Mehdi Hosseini, Faisal Ahmed, Seyed Hossein Hosseini, Sajad Ghafari

**Affiliations:** 1 Department of Urology, School of Medicine, Shiraz University of Medical Sciences, Shiraz, Iran,; 2 Shiraz Geriatric Research Center, Shiraz University of Medical Sciences, Shiraz, Iran,; 3 Shiraz Nephrology-Urology Research Center, Shiraz University of Medical Sciences, Shiraz, Iran,; 4 Urology Research Center, Al-Thora General Hospital, Department of Urology, School of Medicine, Ibb University of Medical Since, Ibb, Yemen

**Keywords:** Percutaneous nephrolithotomy, endourology, urolithiasis, kidney transplant, case report

## Abstract

Urolithiasis is a rare but familiar problem in transplanted kidney patients, with a prevalence rate between 0.23-6.3%. Minimally invasive percutaneous nephrolithotomy (mini-PCNL) is a revised technique that uses a miniature endoscope through a small access sheath and is associated with minor bleeding risk. Only a few cases of mini-PCNL via ultrasonography (US) guidance in transplanted kidneys have been published. We present a 23-year-old female and a 34-year-old man who presented with obstructive uropathy due to impacted stones in their transplanted kidneys. Firstly, the nephrostomy tube was inserted. Then, they underwent mini-PCNL via US guidance. Puncturing the pyelocaliceal system was achieved via a 3.5 MHz US probe. Procedures were performed with a one-shot dilatation technique and a 15-Fr rigid nephroscope. In conclusion, we suggest that if an experienced urologist performs it, the US-guided mini-PCNL is safe and effective in patients with transplanted kidneys.

## Introduction

Urolithiasis is a rare complication of kidney transplantation, with most studies reporting a rate of 0.23-6.3% [1]. Extracorporeal shockwave lithotripsy (ESWL), retrograde intrarenal surgery, and percutaneous nephrolithotomy (PCNL) are minimally invasive options for treating those stones. However, several patients may require more than one technique for stone clearance [1]. The procedure for stones in the transplanted kidney may be particularly challenging due to the patient's immunodeficient state and the kidney's extra-anatomical position [2]. The first mention of the PCNL procedure in a transplanted kidney was made in 1985 [3]. Then, it has been performed at many institutions with satisfactory safety and effectiveness [4-6]. The PCNL procedures in transplanted kidneys are associated with potential graft loss and significant bleeding risk. For that, surgeons believe that these procedures must be restricted to significantly bigger stone burdens and performed only by an experienced urologist and at professional centers [2].

Minimally invasive PCNL (mini-PCNL) is a nominally invasive procedure that uses a small access sheath for puncturing the kidney (Fr 12 to 20). The technique may be accompanied by a minor risk of bleeding and other postoperative complications [2,7]. Here, we report our experience of the mini-PCNL procedure with US guidance in the two cases of urolithiasis in transplanted kidney patients.

## Patient and observation

**Patients information:** the patients were a 23-year-old female and a 34-year-old male with a right iliac fossa allograft renal transplantation history. They were referred to our center due to right lower quadrant pain and decreased urine output last week due to impacted stones in their transplanted kidneys. The patients had a history of consumption of steroids and cyclosporin.

**Clinical findings:** the patients were pale, and they had mild right lower quadrant tenderness.

### Diagnostic assessment

**Figure 1:**
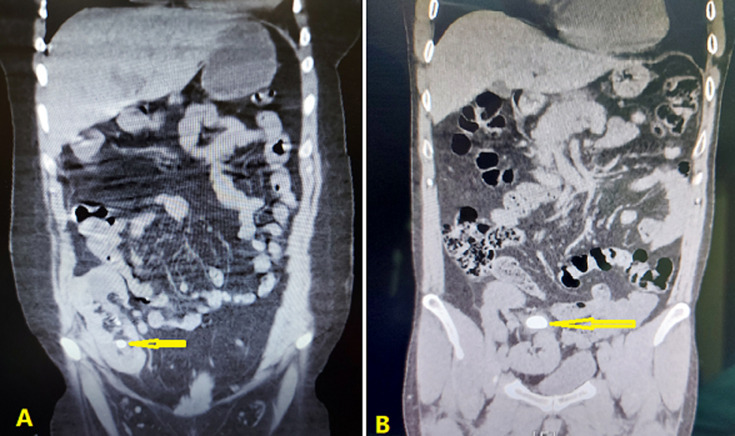
preoperative abdominal pelvic computed tomography (CT) scan showing: A) the lower pole renal stone in the first case (arrow); B) the stone in the renal pelvis in the second case (arrow)

**Case 1 (23-year-old female):** urine analysis confirmed microscopic hematuria (red blood cells 15-20/ high power field), and urine culture was negative. The blood investigation revealed that the serum creatinine was 3.5 mg/dl, blood urea nitrogen (BUN): 45 mg/dl, white blood cells: 11x10^3^/ml, and hemoglobin: 7.4 g/dl. All other investigations were within the normal range. According to her signs and symptoms, ultrasonography (US) was requested that showed a 10×12 mm obstructed stone in the proximal ureter of the transplanted kidney with moderate hydronephrosis that pushed pack to renal lower pole during nephrostomy insertion. A non-contrast abdomen pelvic computed tomography (CT) scan showed a 10x12 mm stone in the lower pole of the transplanted kidney (Figure 1A).

**Case 2 (34-year-old male):** urine analysis confirmed microscopic hematuria (red blood cells 12-20/ high power field), and urine culture was negative. The blood investigation revealed that the serum creatinine: 1.26 mg/dl, blood urea nitrogen (BUN): 14 mg/dl, white blood cells: 9.4x10^3^/ml with average differential count, and hemoglobin: 12.4 g/dl. All other investigations were within the normal range. The US showed a 25x20 mm obstructed stone in the renal pelvis of the transplanted kidney with moderate hydronephrosis. Non-contrast abdomen pelvic CT scan showed the same diagnosis (Figure 1B).

**Therapeutic interventions:** firstly, the nephrostomy was inserted in their kidney one week before the operation. Then expulsive medical therapy with potassium citrate and allopurinol was tried for patients without improvement. After receiving appropriate antibiotics, the options such as ESWL, laparoscopy, and PCNL were discussed with the patients and the patient's families. The benefits and side effects were explained. Finally, the patients chose the mini-PCNL procedure. Both patients were admitted 6 hours before the operation and received parenteral hydration and a single prophylactic antibiotic dose (ceftriaxone 1g). The procedure was performed under general anesthesia. In a supine position, the patients were draped in sterile coverage. The pelvicalyceal system (PCS) was then visualized using the colour Doppler US guide and a 3.5 MHz probe (BK medical). Using a one-shot dilatation technique, an 18G access needle was forwarded into the suitable calyx by attaching the needle to the curved US probe. Then, the stylet was detached, and a 0.035-inch J-tipped guidewire was inserted into the aimed calyx.

**Figure 2:**
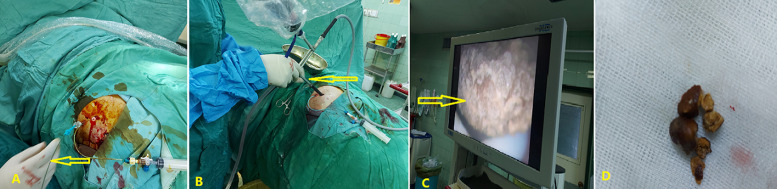
minimally invasive percutaneous nephrolithotomy with ultrasonography guidance showing: A) the J-tipped guidewire insertion into the aimed calyx (arrow); B) the rigid nephroscopy (arrow); C) the stone on the monitor (arrow); D) the removed stones

The skin was incised, and the nephrostomy tract was first dilated with an 8 Fr polyurethane dilator. The Alken guide was then replaced, and a single 18 Fr Amplatz dilator was passed on the Alken guide before inserting an Amplatz sheath into the PCS. We confirmed the proper place using the measured tract length and Amplatz shadow during the insertion. Then, the Amplatz dilator and Alken guide complex were removed, leaving the Amplatz sheath and working guidewire in place. A 15 Fr rigid nephroscopy followed the procedure. Throughout the renal access, all processes were monitored under the supervision of the US without any need for fluoroscopy. The stone was fragmented by lithoclast and extracted with forceps. Warm saline solution was performed as irrigation to avoid hyponatremia and hypothermia. The US confirmed the operation's stone-free status at the end [8,9]. The previous nephrostomy tube was left at the end of the procedure (Figure 2). The total operative time was about 76 minutes.

**Follow-up and outcomes:** the patient's post-surgical recovery was unremarkable in both patients. The day after the operation, a plain abdominal film (KUB X-ray) and the US were performed to rule any residual stone. The urethral Foley was removed after 12 to 24 hours. Both patients were discharged with oral antibiotics. The nephrostomy tubes were removed on the second postoperative week.

**Patient perspective:** the patients were happy with the successful outcome of the surgery.

**Informed consent:** a written informed consent was obtained from the patients for participation in our study.

## Discussion

Conventional PCNL procedure for treating stones in the transplanted kidneys has been a well-accepted therapeutic intervention explained by numerous surgeons over the last decade [1,6,8]. However, information regarding mini-PCNL for transplanted kidneys is limited, with few reports. For that, we reported our experiences of mini-PCNL procedure via US guidance in two transplanted cases without any complications. In transplanted kidney patients with obstructive uropathy, the primary aim is to resolve the obstruction and perform definitive therapy [10]. For that, we first inserted the nephrostomy; then, we achieved the mini-PCNL after one week as definitive therapy.

Previous papers on mini-PCNL included seven patients by He *et al*. treated over five years period, a subset of 8 patients by Rifaioglu *et al*. in North America over ten years, a case report by Munk *et al*. and a case reported by Kadlec and colleagues [1,2,4,11]. A new study explains five antegrade ureteroscopic surgeries done on patients with kidneys transplantation; however, no tract dilation was conducted, and the stone size was about 15 mm [5]. In our cases, the puncture of the PCS was achieved using a 3.5 MHz US probe; the procedure was performed using a one-shot dilatation technique and via a 15 Fr rigid nephroscope.

In our cases, the stone size (12 and 25 mm) and location (lower pole and renal pelvis) were significantly sufficient that PCNL was an appropriate choice, and nominal tract dilation was preferred to reduce the risk of bleeding, which was in agreement with a previous report [2,10]. Reviewing another option for stone in the transplanted kidney, it was mentioned that the retrograde ureteroscopic intrarenal procedure necessitated a ureteroneocystostomy creation in the bladder dome, which makes finding and cannulating the ureteral orifice hard; also, the irrigation in an impeded system may result in further infection. Open nephrolithotomy is not advised due to the danger of more infection, severe adhesion, wound infection, and delayed wound healing process. The ESWL was suggested for intermediate stone size (0.5-1.5 cm) with the prone position. However, due to the location of the kidney over the bony pelvis, identifying such stones can be challenging. The clearance rate of fragmented stone can be restricted, particularly for the lower pole calculi. Remnant fragments pose a risk of infection and can act as a breeding ground for new stones. When ESWL is performed, Stein Strasse can be problematic due to the difficult identification of the ureteral orifice [1].

Mini-PCNL is performed using small percutaneous tract sheaths (Fr 11-20). Additionally, mini-PCNL has the advantages of less blood loss, enhanced intrarenal flexibility, significantly reduced postoperative pain, and a shorter hospital stay in compression to conventional PCNL. One of the limitations is the need to disintegrate stones into small enough fragments to match up through a smaller sheath, which results in extended operative times [10]. In our cases, we used US-guided mini-PCNL. The advantages of US-guided mini-PCNL include continuous monitoring of the deeper structures and vessels during the operation, accurate estimations in access to the stone, no exposure to radiation to the staff, and no need for contrast injection [8]. Because the tract to the collecting system is shorter in pediatric and adolescent cases than in adults, the US facilitates safer tract dilation and exact needle placing to the PCS [8].

## Conclusion

We suggest that, if performed by an experienced urologist, the US-guided mini-PCNL technique is harmless and could be a good option in patients with transplanted kidneys.

## References

[1] He Z, Li X, Chen L, Zeng G, Yuan J (2007). Minimally invasive percutaneous nephrolithotomy for upper urinary tract calculi in transplanted kidneys. BJU Int.

[2] Kadlec AO, Ross MJ, Milner JE (2013). Mini-percutaneous nephrolithotomy with ureteral access sheath in a transplanted kidney: case report and literature review. Urol Int.

[3] Hulbert JC, Reddy P, Young AT, Hunter DW, Castaneda-Zuniga W, Amplatz K (1985). The percutaneous removal of calculi from transplanted kidneys. J Urol.

[4] Rifaioglu MM, Berger AD, Pengune W, Stoller ML (2008). Percutaneous management of stones in transplanted kidneys. Urology.

[5] Hyams E, Marien T, Bruhn A, Quirouet A, Andonian S, Shah O (2012). Ureteroscopy for transplant lithiasis. J Endourol.

[6] Stravodimos KG, Adamis S, Tyritzis S, Georgios Z, Constantinides CA (2012). Renal transplant lithiasis: analysis of our series and review of the literature. J Endourol.

[7] Chan DY, Jarrett TW (2000). Mini-percutaneous nephrolithotomy. J Endourol.

[8] Eslahi A, Ahmed F, Hosseini MM, Rezaeimehr MR, Fathi N, Nikbakht HA (2021). Minimal invasive percutaneous nephrolithotomy (Mini-PCNL) in children: ultrasound versus fluoroscopic guidance. Arch Ital Urol Androl.

[9] Ahmed F, Askarpour MR, Eslahi A, Nikbakht HA, Jafari SH, Hassanpour A (2018). The role of ultrasonography in detecting urinary tract calculi compared to CT scan. Res Rep Urol.

[10] Markic D, Krpina K, Ahel J, Grskovic A, Spanjol J, Rubinic N (2016). Treatment of kidney stone in a kidney-transplanted patient with mini-percutaneous laser lithotripsy: a case report. Case Rep Nephrol Dial.

[11] Munk AK, Seif C, Renders L, Jünemann KP, Braun PM (2007). Percutaneous nephrolithotomy of an upper pole calix stone in a transplanted kidney. Aktuelle Urol.

